# Single-cell reconstruction of follicular remodeling in the human adult ovary

**DOI:** 10.1038/s41467-019-11036-9

**Published:** 2019-07-18

**Authors:** X. Fan, M. Bialecka, I. Moustakas, E. Lam, V. Torrens-Juaneda, N. V. Borggreven, L. Trouw, L. A. Louwe, G. S. K. Pilgram, H. Mei, L. van der Westerlaken, S. M. Chuva de Sousa Lopes

**Affiliations:** 1 Department of Anatomy and Embryology, Leiden University Medical Center, 2333 ZC Leiden, Netherlands; 2 Sequencing Analysis Support Core, Leiden University Medical Center, 2333 ZC Leiden, Netherlands; 3 Department of Immunohematology and Blood Transfusion, Leiden University Medical Center, 2333 ZA Leiden, Netherlands; 4 Department of Gynaecology, Division of Reproductive Medicine, Leiden University Medical Center, 2333 ZA Leiden, Netherlands; 5 Department for Reproductive Medicine, Ghent University Hospital, 9000 Ghent, Belgium

**Keywords:** Mechanisms of disease, Functional clustering

## Abstract

The ovary is perhaps the most dynamic organ in the human body, only rivaled by the uterus. The molecular mechanisms that regulate follicular growth and regression, ensuring ovarian tissue homeostasis, remain elusive. We have performed single-cell RNA-sequencing using human adult ovaries to provide a map of the molecular signature of growing and regressing follicular populations. We have identified different types of granulosa and theca cells and detected local production of components of the complement system by (atretic) theca cells and stromal cells. We also have detected a mixture of adaptive and innate immune cells, as well as several types of endothelial and smooth muscle cells to aid the remodeling process. Our results highlight the relevance of mapping whole adult organs at the single-cell level and reflect ongoing efforts to map the human body. The association between complement system and follicular remodeling may provide key insights in reproductive biology and (in)fertility.

## Abstract (web summary)

In the ovary, follicular degeneration occurs after folliculogenesis, with one dominant follicle reaching maturity every month. Here, by performing single cell RNA-seq of adult human follicles, the authors identify subclasses of granulosa and theca cell lineages, which correlate with the growth or degeneration of the follicles.

## Introduction

In the absence of a pregnancy, both the ovary and the uterus undergo significant monthly remodeling during the entire reproductive period (about 40 years) in healthy women. From the 1 million follicles present in the ovary at birth, only about 500 reach the ovulatory phase during the reproductive span of healthy women, while the rest degenerates^1–3^. Follicular growth involves: (1) maturation of the oocyte; (2) the extensive cellular proliferation and differentiation of the granulosa cells (GC) to support the oocyte (cumulus GC) and allow the accumulation of follicular fluid in the antrum (mural GC); and (3) the generation of a specialized tissue layer of theca cells (TC) from stromal cells, surrounding the follicle, with high vascularization^2–4^.

Follicular degeneration or atresia occurs at any stage of folliculogenesis^4,5^. As per month only one dominant follicle reaches the ovulatory stage^6^, it is imperative for ovarian homeostasis that other growing follicles are efficiently removed by atresia to accommodate following waves of follicular growth. Robust tissue remodeling also occurs monthly with the transformation of the ovulatory follicle into the hormone-producing corpus luteum (formed by lutein GC, lutein TC, and vasculature), followed by regression to a corpus albicans^2–5^. Although the molecular signature of the cumulus GC of the dominant ovulatory follicle is known, due to the accessibility to material from patients using artificial reproduction technologies^7,8^, the continuous process of follicular growth and degeneration is not well understood in humans. Here, we have thought to identify the somatic cell types and associated signals that regulate tissue remodeling in the adult ovary. Understanding these mechanisms is paramount to pinpoint causes of infertility and to develop both treatments and disease models^1,9–11^.

## Results

### Sample preparation from the inner cortex of adult ovary

**Fig. 1:**
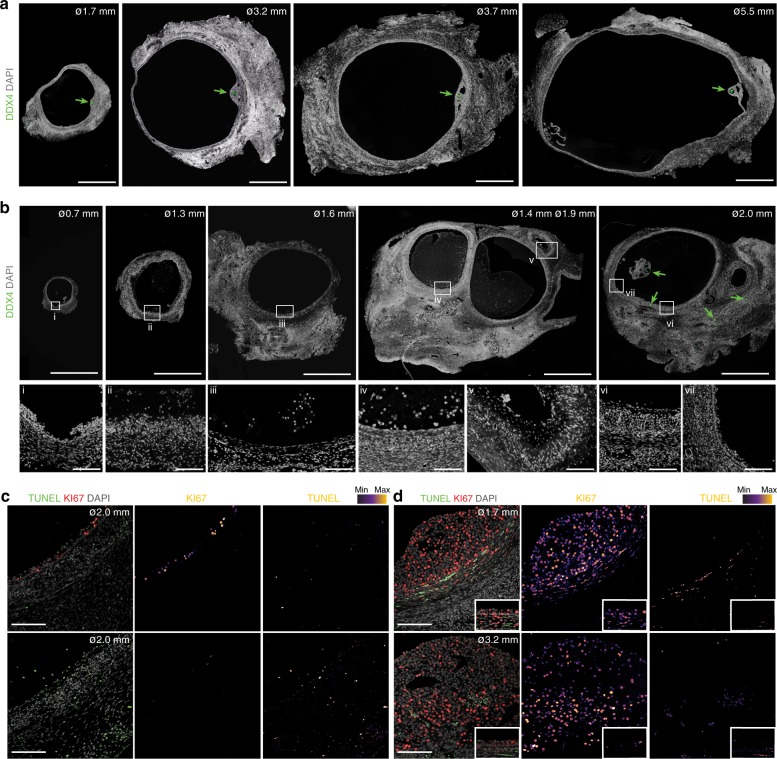
Morphology of different follicles in adult ovaries. **a**, **b** Immunofluorescence of several healthy (**a**) and atretic follicles (**b**) of different sizes present in the ovaries immunostained for DDX4. Green arrows point to (DDX4-positive) oocytes. Panels in the bottom row show magnifications of the boxed areas with corresponding roman numbers. Tissue was counterstained with DAPI. Scale bars are 1 mm in two top rows and 100 μm in bottom row (panels i-vii). **c**, **d** Immunofluorescence of atretic (**c**) and healthy follicles (**d**) of different sizes present in the ovaries analyzed for KI67 and TUNEL. Insets in **d** show a different area of the follicle showing mural GC with same magnification. Single channel images were converted to an intensity map. Scale bars are 100 μm

We have analyzed anonymised ovarian tissue (inner cortex) from adult women (*N* = 5) undergoing fertility preservation procedures (outer cortex is cryopreserved). This included several small antral follicles (tissue with an outer diameter of about 2–4 mm including stroma, TC, and a visible follicle of 1–2 mm in diameter) and selectable follicles (tissue with an outer diameter of about 5–8 mm including stroma, TC, and a visible follicle with 2–5 mm of diameter) (Fig. 1a, b). Follicles in (early) stages of atresia (Fig. 1b) may resemble growing follicles (Fig. 1a) in size, but the cellular organization of mural GC and TC layers differ. Follicles in (early) stages of atresia (Fig. 1c) showed less cellular proliferation and only moderate levels of apoptosis compared to growing follicles (Fig. 1d). In these (early) atretic follicles, the layer of TC showed pronounced signs of luteinization (hypertrophied morphology), whereas the GC detach from the basement membrane (Fig. 1b, iv and vi)^12,13^. In later stages of atresia, it remains unclear whether TC differentiate to or are replaced by fibroblasts (Fig. 1b, v)^12,13^.

**Fig. 2:**
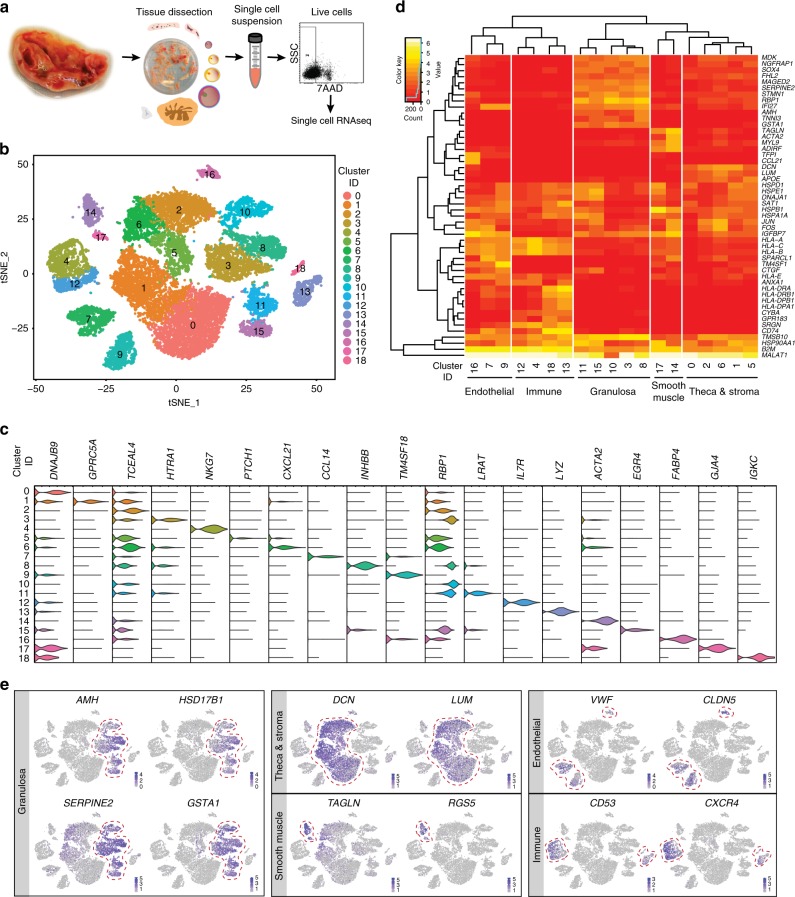
Transcriptome map of human adult ovaries analyzed. **a** Schematic representation of human ovarian tissue preparation for single cell transcriptome analysis. **b** tSNE cluster map revealing 19 specific clusters representing the major ovarian somatic cell types. **c** Violin plots showing expression of one representative differential expressed gene for each cluster. **d** Heatmap and hierarchical clustering based on expression of top 50 most variable genes. **e** tSNE cluster map showing expression of genes characteristic the major ovarian somatic cell types. Red dashed lines give the boundaries of the main clusters of interest

A total of 31 different tissue samples from the inner cortex of those adult ovaries (*N* = 5), including stroma and visible follicles of different sizes were collected for single-cell sequencing (Supplementary Data 1). After enzymatic dissociation, each sample was subjected to FACS-sorting to remove dead cells (Fig. 2a) and analyzed by single-cell sequencing using the 10X Genomics platform (56,206 cells) (Supplementary Data 1). The data was filtered using quality control parameters as described in R package Seurat^14^ (Supplementary Fig. 1a). In addition, cells expressing high levels (>6% of total UMIs) of dissociation-related genes^15^ were excluded from further analysis (Supplementary Fig. 1b; Supplementary Data 1). We retained 20,676 cells expressing 2516 (highly variable) genes for further analysis.

### Single cell clustering and cell type identification

Using a Seurat-based workflow and a correction step to minimize patient-specific effects (using mutual nearest-neighbor method), we clustered the cells and identified 19 clusters. In the same workflow, we run the non-linear dimensionality reduction algorithm tSNE to visualize the cells in a two-dimensional plot (Fig. 2b; Supplementary Fig. 1c, d). We calculated the top 30 differentially expressed genes (DEGs) from each cluster, filtered for genes with average log_e_ fold change > 0.5, sorted by their adjusted *p*-values (Wilcoxon rank sum test) (Supplementary Data 2) and plotted one representative DEG per cluster (Fig. 2c). Based on the DEGs, we performed a Gene Ontology (GO) enrichment analysis (Supplementary Data 3) to facilitate cluster identification. Next, we generated a hierarchical clustering using the 50 most variably expressed gene means per cluster and distinguished five major cell types: GC (five clusters), TC and stroma (five clusters), smooth muscle cells (two clusters), endothelial cells (three clusters), and immune cells (four clusters) (Fig. 2d). To confirm the identity of these cell types in the tSNE, we colored the single cells according to the expression levels of several expected marker genes: *AMH*, *HSD17B1*, *SERPINE2*, *GSTA1* for GC; *DCN*, *LUM* for TC and stroma; *TAGLN* and *RGS5* for smooth muscle cells; *VWF* and *CLDN5* for endothelial cells; and *CD53* and *CXCR4* for immune cells (Fig. 2e).

To provide a brief characterization of the cells removed from the total dataset (56,206 cells), we plotted the retained cells (20,676 cells) in a tSNE that included all cells (Supplementary Fig. 1e–g). Instead of 19 clusters, we obtained 21 clusters, each containing both retained and removed cells. Comparing the DEGs associated with each of the 21 clusters (Supplementary Data 4) with the DEGs associated with the 19 clusters obtained from the retained cells (Supplementary Data 2), we were able to match the large majority of the clusters, confirming that the cells removed from each cluster corresponded to stressed cells from each specific cluster, due to high levels of dissociation-related genes^15^. From the DEGs of the unmatched clusters, we were able to identify those extra populations as stroma and endothelial cells, related to the retained stroma and endothelial cell clusters. We cannot exclude that those correspond to biological relevant populations.

### Vascular remodeling in the adult ovary

**Fig. 3:**
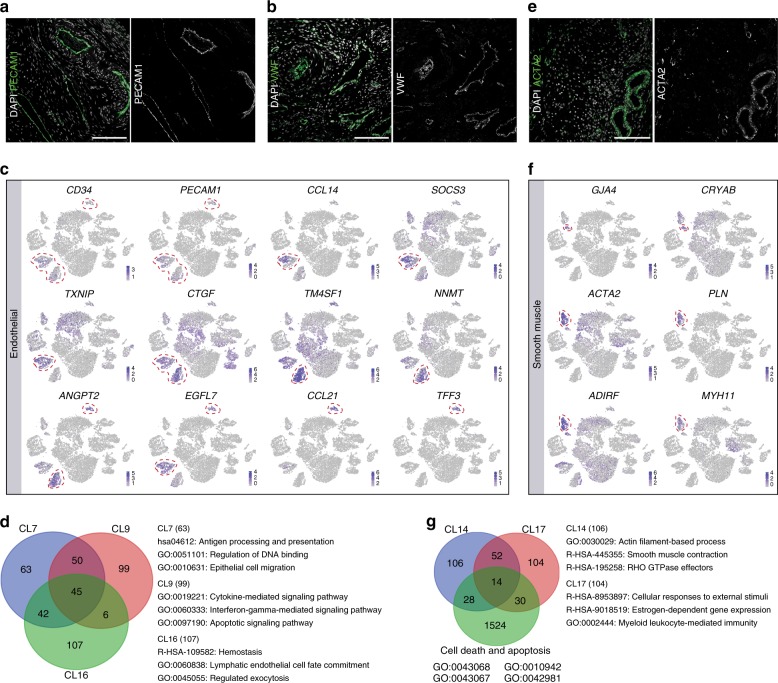
Vascular remodeling in the ovaries analyzed. **a**, **b** Immunofluorescence of ovarian stroma for PECAM1 **a**, VWF **b**, and the respective single channel images. Slides were counterstained with DAPI. Scale bars are 100 μm. **c** tSNE cluster maps showing expression of selected endothelial marker genes. Red dashed lines give the boundaries of the endothelial-clusters of interest. **d** Venn diagram showing the intersection of 200 differential expressed genes (DEGs) of the three endothelial cell clusters (CL7, CL9, and CL16); and three selected enriched terms obtained for the unique DEGs. **e** Immunofluorescence of ovarian stroma for ACTA2, and the respective single channel image. Slides were counterstained with DAPI. Scale bars are 100 μm. **f** tSNE cluster map showing expression of selected smooth muscle marker genes. Red dashed lines give the boundaries of the smooth muscle-clusters. **g** Venn diagram showing the intersection of 200 differential expressed genes (DEGs) of the two smooth muscle cell clusters (CL14 and CL17) and genes from four-cell death and apoptosis-related GO terms (GO:0043068 positive regulation of programmed cell death, GO:0010942 positive regulation of cell death, GO:0043067 regulation of programmed cell death, GO:0042981 regulation of apoptotic process); and three selected enriched terms obtained for the unique DEGs

Vascular remodeling in the ovary, supporting the dynamic changes in follicular growth and degeneration, has gained more attention in recent years^2–4^. We identified three separate clusters (CL) of endothelial cells (CL7, CL9, CL16) expressing markers associated with lymph and blood vascular system (such as *PECAM1*, *CD34*, *CTGF*), but also associated with remodeling and inflammatory response (such as *TXNIP*, *ANGPT2*) (Fig. 3a–d). The DEGs of CL7 (such as *CCL14*, *SOCS3*, *EGFL7*) and CL16 (such as *CCL21*, *TFF3*) are linked to angiogenesis and lymphatics, respectively, while DEGs of CL9 (*TM4SF1*, *NMMT*) were more related to regulation of apoptosis (Fig. 3c, d). The clusters of smooth muscle cells (CL14, CL17) also showed features of growth and remodeling: many DEGs of CL17 (such as *CRYAB*, *GJA4*) were involved in regulation of immune response and apoptosis, whereas DEGs of CL14 (such as *ACTA2*, *PLN*, *ADIRF,* and *MYH11*) associated with mature smooth muscle cells (Fig. 3e–g).

### Molecular and cellular signature of different GC populations

**Fig. 4:**
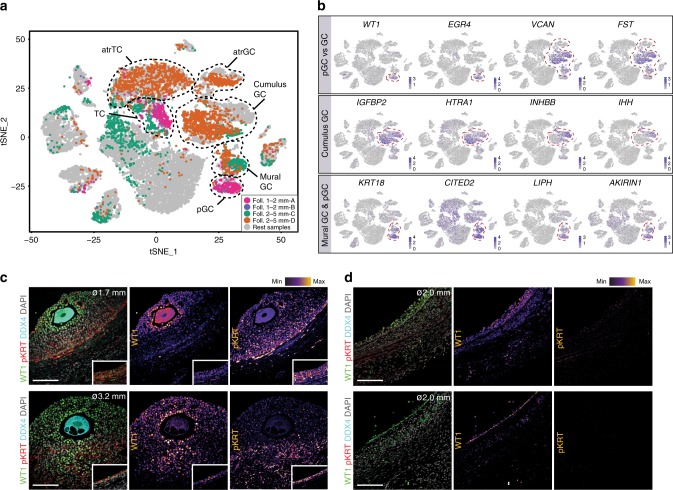
Divergent populations of granulosa cells in different follicles. **a** Distribution of single cells from different-sized follicles on the tSNE. Black dashed lines give the boundaries of several clusters of GC (common progenitor GC (pGC), mural GC, cumulus GC, atretic GC (atrGC), theca cells (TC), and atretic TC (atrTC)). **b** tSNE cluster map showing expression of selected marker genes differentially expressed by GC and pGC (top row), cumulus GC (middle row), and mural GC and pGC (bottom row). Red dashed lines give the boundaries of the GC-clusters of interest. **c**, **d** Immunostaining of follicles (ø, diameter) growing (**c**) and atretic (**d**) for WT1 and pan-KRT (pKRT). Inset shows mural GC of the same follicle with the same magnification. Single channel images were converted to an intensity map. Scale bars are 100 μm

To reveal the dynamic cellular changes that take place during antral follicle maturation, we focused first on the analysis of the GC in antral follicles (Fig. 4a; Supplementary Fig. 2a, b). The GC of small antral follicles (1–2 mm diameter) clustered pronouncedly in cluster (CL15) showing *WT1*^high^/*EGR4*^high^/*VCAN*^low^/*FST*^low^ expression (Fig. 4b), suggesting that at that stage mural and cumulus GC still have a common progenitor (pGC) signature. In agreement WT1 was expressed in GC, in particular in the GC forming the corona radiata (Fig. 4c), in contrast to mouse ovaries where WT1 marked TC-progenitors^16^. GC from selectable follicles (2–5 mm diameter) separated into two main cell types: cumulus GC (*VCAN*^high^/*FST*^high^/*IGFBP2*^high^/*HTRA1*^high^/*INHBB*^high^/*IHH*^high^) and mural GC (*WT1*^low^/*EGR4*^low^/*KRT18*^high^/*CITED2*^high^/*LIHP*^high^/*AKIRIN1*^high^) (Fig. 4b)^7,17^. In agreement, pan-KRT immunostaining revealed higher protein expression in mural than in cumulus GC (Fig. 4c).

**Fig. 5:**
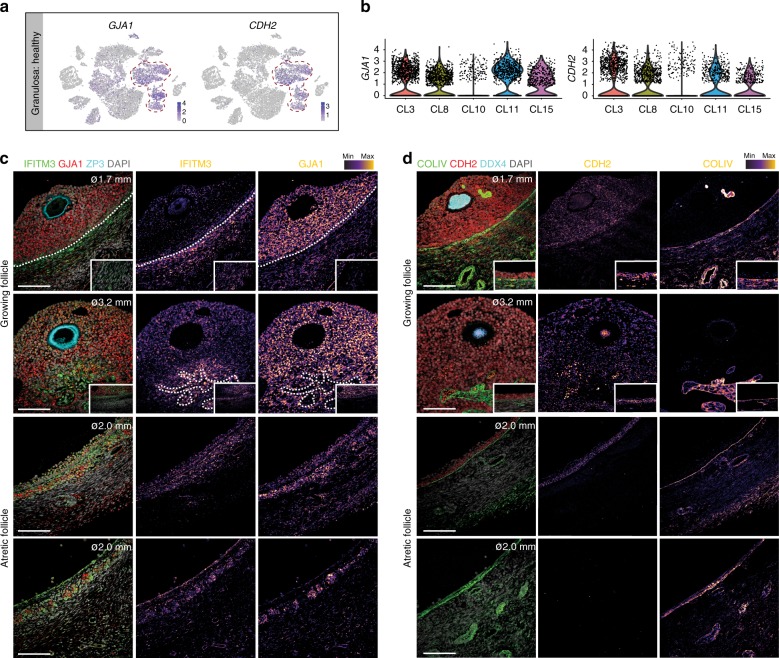
Granulosa cells in early atretic follicles. **a** tSNE cluster map showing expression of selected genes downregulated in CL10, but not on the other granulosa cell (GC) clusters. Red dashed lines give the boundaries of expression. **b** Violin plots showing expression levels of *GJA1* and *CDH2* in the different clusters of GC. **c** Immunostaining of follicles (ø, diameter) growing (top two rows) and atretic (bottom two rows) for IFITM3, GJA1, and ZP3. Inset shows mural GC of the same follicle with same magnification. Single channel images were converted to an intensity map. White dotted line marks the basement membrane. Scale bars are 100 μm. **d** Immunostaining of follicles (ø, diameter) growing (top two rows) and atretic (bottom two rows) for CDH2, COLIV, and DDX4. Inset shows mural GC of the same follicle with same magnification. Single channel images were converted to an intensity map. Scale bars are 100 μm

Several samples (such as selectable follicle D, but not follicle C) contained GC in CL10 (Fig. 4a, Supplementary Fig. 2a, b). The GC of CL10 were negative for several GC markers, such as *VCAN* and *FST* (Fig. 4b), but were also negative for *KRT18* (Fig. 4b), similarly to pan-KRT-negative GC in atretic follicles (Fig. 4d). This suggested that CL10 could represent GC in the early stages of atresia. The GC in CL10 expressed lower levels of *GJA1* and *CDH2* compared to the other GC clusters (Fig. 5a, b). Lower levels of GJA1 have been described in GC of atretic compared with healthy follicles in rats^18^, where it was suggested that reduced gap junctions, and hence cellular communication, play a role in atresia. Using immunostaining, we confirmed lower expression of GJA1 and CDH2 in GC of atretic follicles in humans (Fig. 5c, d, bottom two rows) compared with growing follicles (Fig. 5c, d, top two rows).

**Fig. 6:**
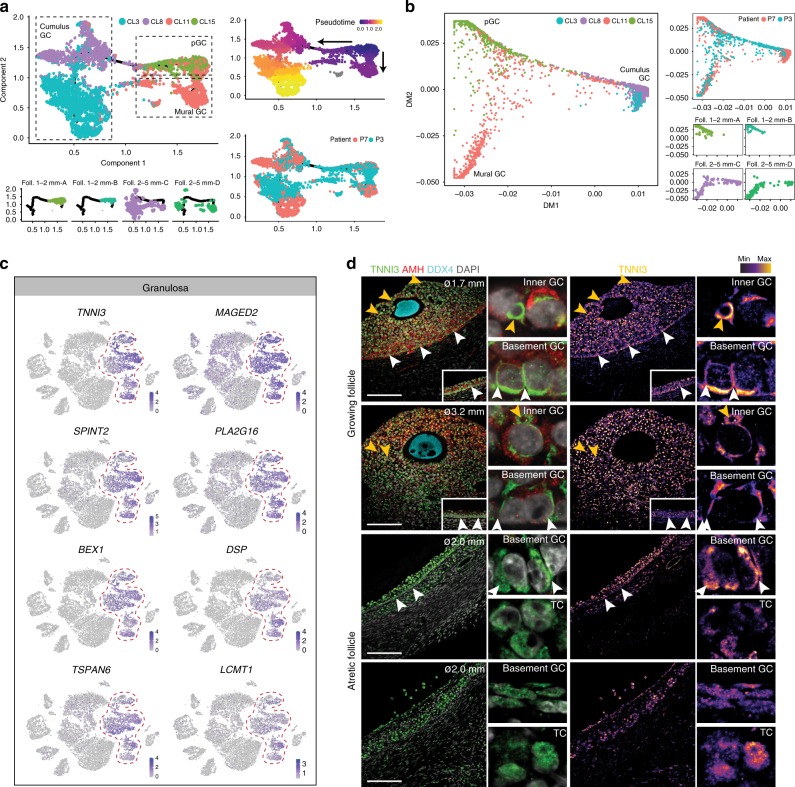
Cell trajectory analysis and characterization of granulosa cells. **a**, **b** Analysis of cell trajectories of granulosa cells (GC) (CL3, CL8, CL11, CL15) by Monocle **a** and Diffusion maps **b**. Individual cells (dots) are colored by cluster, follicle, patient, and pseudotime (Monocle). **c** tSNE cluster map showing selected genes expressed by GC. Red dashed lines give the boundaries of the GC-clusters. **d** Immunostaining of follicles (ø, diameter) growing (top two rows) and atretic (bottom two rows) for TNNI3, AMH, and DDX4. Higher magnification of inner and basement GC is shown on the right side. Single channel images for TNNI3 converted to an intensity map are shown. White arrowheads depict basement GC, yellow arrowheads depict inner GC. Scale bars are 100 μm

We used two independent methods to analyze the cell trajectories of the GC. Due to the limited number of samples available, the intermediate states are not well represented and hence our conclusions regarding trajectories should be considered preliminary. Pseudotime analysis using Monocle 3 alpha, that places the progenitor cell population in the middle of a longer trajectory segment, revealed that pGC (CL15) branched to mural GC (CL11) and mature cumulus GC (CL8 and CL3) (Fig. 6a). As pseudotime analysis is susceptible to be affected by inter-individual variation, we highlighted cells from two individuals (P7 and P3) showing cells of both in each cluster. The cell trajectories obtained by Monocle 3 alpha were consistent with the cell trajectories obtained using Diffusion maps (Fig. 6b).

Several genes not earlier associated with GC include *TNNI3*, *MAGED2*, *SPINT2 PLA2G16*, *BEX1*, *DSP*, *TSPAN6,* and *LCMT1* (Fig. 6c). Due to the limited amount of samples per individual, we cannot exclude that some genes may represent differential expression between individuals. We verified the expression of TNNI3 in GC and observed a specific cellular localization. This may be important for regulating GC shape and function: TNNI3 formed a characteristic ring-shaped structure in inner GC, but was distributed to the baso-lateral cell membrane of the GC on the basement membrane (Fig. 6d).

### Molecular and cellular signature of TC and stromal cells

**Fig. 7:**
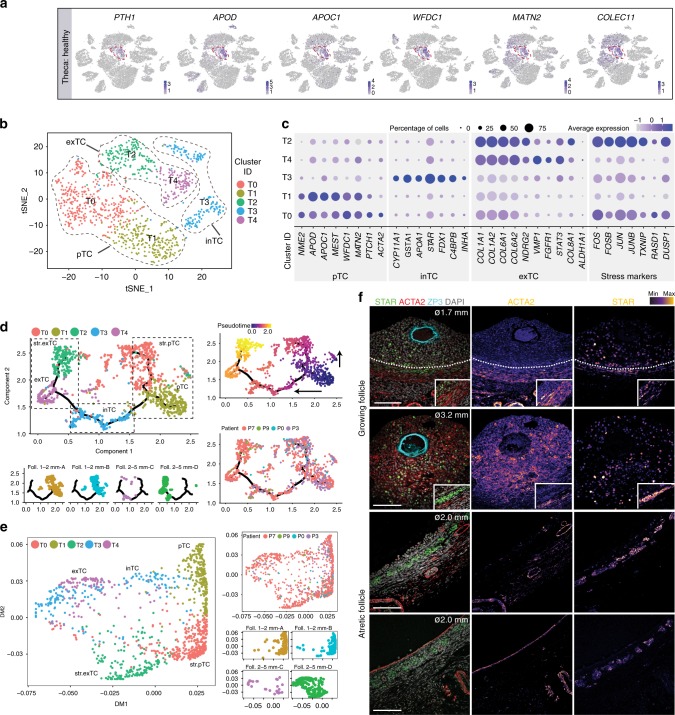
Divergent populations of theca cells in different follicles. **a** tSNE cluster map showing expression of selected theca cells (TC) genes. Red dashed lines give the boundaries of the expression. **b** tSNE cluster map revealing sub-clusters of CL5 representing ovarian TC types. Black dashed lines give the boundaries of several sub-clusters of TC: common progenitor TC (pTC), externa TC (exTC), and interna TC (inTC). **c** Expression of marker genes in sub-clusters of CL5. **d**, **e** Analysis of cell trajectories of TC (CL5) by Monocle **a** and Diffusion maps **b**. Individual cells (dots) are colored by cluster, follicle, patient, and pseudotime (Monocle). **f** Immunostaining of follicles (ø, diameter) growing (top two rows) and atretic (bottom two rows) for STAR, ACTA2, and ZP3. Inset shows mural GC of the same follicle with same magnification. Single channel images were converted to an intensity map. White dotted line marks the basement membrane. Scale bars are 100 μm

The TC appeared in three separate TC clusters (CL2, CL5, CL6) (Figs. 2b, 4a). Those included TC present in samples with follicles (Supplementary Fig. 2a, b) and in stromal samples without visible follicles, but that could contain TC from follicle walls, corpus luteum or albicans (Supplementary Fig. 2c). TC from (healthy) follicles, such as follicles A, B, and C, were mainly clustered in CL5 (Fig. 4a), characterized by expression of known markers *PTH1*, *APOD*, *APOC1*, and several genes not earlier associated with TC, such as *WFDC1*, *MATN2*, *COLEC11* (Fig. 7a). TC from the small antral follicles A and B did not overlap with TC from selectable follicle C in CL5 (Fig. 4a). To explore differences between these domains inside CL5, we calculated a separate tSNE using only CL5 cells to further characterize the sub-populations of TC (Fig. 7b). Combining differential gene expression of the obtained TC sub-clusters (Fig. 7c; Supplementary Data 5) with cell trajectory analysis using two independent methods (Monocle 3 alpha and Diffusion maps) (Fig. 7d, e), we concluded that TC from the small follicles correspond to common progenitor TC (pTC) that progress to interna TC (inTC) and externa TC (exTC) from selectable follicles. Both CL-T0 and CL-T1 showed a profile of pTC, but cells in CL-T0 showed additional expression of stress-related markers (such as *FOS*, *JUN*) (Fig. 7c). Similarly, CL-T2 and CL-T4 showed a profile of exTC, but cells in CL-T2 also showed more pronounced expression of stress-related markers (such as *FOS*, *JUN*) (Fig. 7c). Interestingly, there were differences in the trajectories obtained by the two methods regarding the localization of CL-T2 (stressed exTC) in the trajectory. In monocle, we obtained pTC → stressed pTC + pTC → inTC → exTC → stressed exTC (Fig. 7d). By contrast, in the diffusion maps the two population of stressed TC (CL-T0 and CL-T2) were in the same trajectory and we observed pTC → stressed pTC → stressed exTC + pTC → inTC → exTC (Fig. 7d).

Immunostaining for STAR and ACTA2 was sufficient to distinguish inTC from exTC in growing follicles (Fig. 7f, top two rows). This highlights similarities between TC and the corresponding male-equivalent (ACTA2+) peritubular myoid cells in the testis^19^. In atretic follicles, STAR expression remained in TC, whereas ACTA2 became more restricted to smooth muscle cells (Fig. 7f, bottom two rows). IFITM3, so far only reported in bovine follicles^20^ proved useful in humans to distinguish (IFITM3+) TC from (GJA1+) GC at least in growing follicles (Fig. 5c). Surprisingly, we observed in two follicles from one woman (and not in any of the follicles analyzed from the other four women) that (CDH2−/COLIV+ or GJA1−/IFITM3+) TC showed clear protrusions into the cumulus (CDH2+/COLIV− or GJA1+/IFITM3−) GC area (Fig. 5c, d). Due to the low number of individual women (*N* = 5), it remains unclear how common this feature is.

**Fig. 8:**
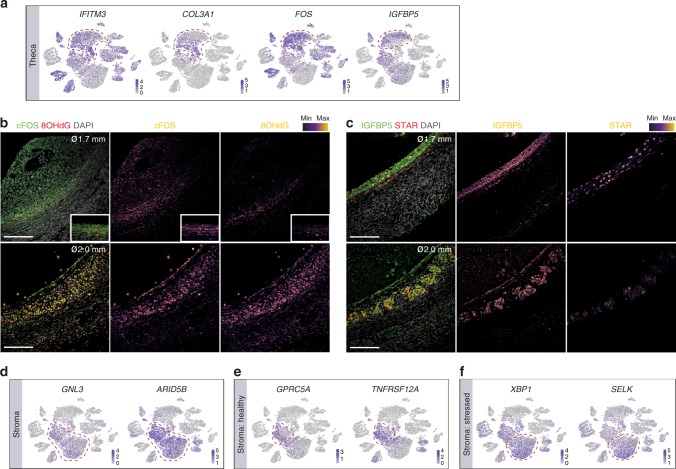
Characteristics of theca and stromal cells. **a** tSNE cluster map showing expression of selected marker genes healthy and atretic theca cells (TC). Red dashed lines give the boundaries of the TC-clusters of interest. **b**, **c** Immunostaining of follicles (ø, diameter) growing (top row) and atretic (bottom row) for cFOS and 8OHdG (**b**) and for IGFBP5 and STAR **c**. Inset shows mural GC and TC of the same follicle with same magnification. Single channel images were converted to an intensity map. Scale bars are 100 μm. **d**, **f** tSNE cluster map showing expression of selected marker genes in the ovarian stroma **d**, healthy stroma **e** and stressed stroma (**f**). Red dashed lines give the boundaries of the stromal clusters of interest

TC from (early atretic) follicle D and present in stromal samples were mainly present in CL2 and CL6 (Fig. 4a, Supplementary Fig. 2c), suggesting that those may represent atretic TC. The TC in CL2 and CL6 expressed *IFITM3*, lower levels of *COL3A1* and higher levels of *FOS* and *IGFBP5* compared to the TC in CL5 (Fig. 8a). Using immunostaining, we confirmed expression of FOS and IGFBP5 in the TC of early atretic follicles, where it colocalized with marker of DNA/RNA damage 8OHdG and STAR (Fig. 8b, c, bottom row) in contrast to TC in growing follicles (Fig. 8b, c, top row).

Most stromal cells from selectable follicle C clustered in CL1 (Fig. 3a). Although stromal clusters (CL0, CL1) showed high expression of *GNL3* and *ARID5B* (Fig. 8d), CL0 expressed high levels of *XBP1* and *SELK* (Fig. 8e), both involved in endoplasmic reticulum (ER)-stress-induced apoptosis, whereas CL1 expressed high levels of *GPRC5A* and *TNFRS12A* (Fig. 8f).

### Immune cells and complement system in the adult ovary

**Fig. 9:**
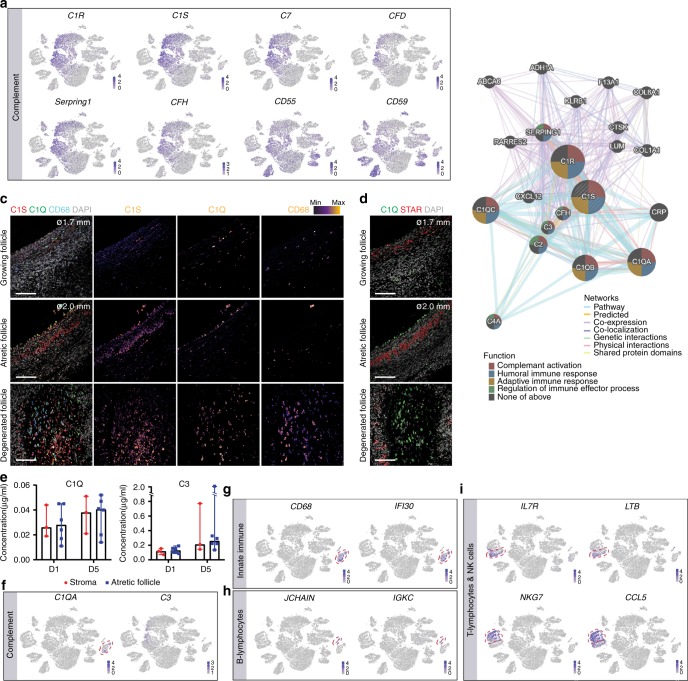
Complement and immune system in the adult ovaries. **a** tSNE cluster map showing expression of selected complement genes. **b** Gene network of C1S in TC and stroma. The color of the circles represents function and the color of the edges represent networks. **c** Immunostaining of follicles (ø, diameter) growing (top row), atretic (middle row), and degenerated (bottom row) for C1S, C1Q, and CD68. Single channel images were converted to an intensity map. Scale bars are 100 μm. **d** Immunostaining of follicles (ø, diameter) growing (top row), atretic (middle row), and degenerated (bottom row) for C1Q and STAR. Scale bars are 100 μm. **e** Concentration of secreted C1Q and C3 produced by pieces of human ovarian stroma (*N* = 3 samples) and atretic follicle walls (*N* = 6 samples) after 1 and 5 days of culture. Median and sample distribution (dots) are shown. **f** tSNE cluster map showing expression of *C1QA* and *C3*. **g–i** tSNE cluster map showing expression of selected immune marker genes for innate immune cells **g**, B lymphocytes **h** and T lymphocytes and NK cells **i**. Red dashed lines give the boundaries of the specific immune-clusters

Interestingly, both the TC clusters (CL2, CL5, CL6) and stroma (CL1) showed pronounced expression of several components of the complement system, such as C1R, C1S, and C7 (Fig. 9a). In agreement, gene network in CL1, CL2, CL5, and CL6 also revealed an association between complement genes and TC and stromal genes (such as *LUM*, *COL6A1*, *COL1A1*) (Fig. 9b). On the other hand, pGC, mural GC, and other cell populations in the ovary expressed *CD55* and *CD59*, known to protect against targeting and damage by the complement cascade (Fig. 9a), suggesting a concerted mechanism involving the complement system to allow growth and degeneration of the different follicular compartments in a timely manner.

The complement system has been reported in the follicular fluid of women undergoing in vitro fertilization (IVF) treatment^21^, but has not been associated with physiological tissue remodeling in the human adult ovary. Using immunofluorescence, we confirmed increasing expression of C1S from healthy to degenerating follicles (Fig. 9c, d), whereas expression of C1Q was mainly confined to (CD68+) macrophages^22^ present in in the ovary. There was an overabundance of (CD68+) macrophages in degenerated follicles (Fig. 9c, d), suggesting an innate immune response during follicular remodeling. Although most circulating complement proteins (except C1Q and C7) are produced in the liver, local production of complement has been detected in a variety of tissues and cells^23^. To determine the local production of complement components by ovarian cells, we cultured pieces of human ovarian stroma (*N* = 3) and atretic follicle walls (*N* = 6) for 1 and 5 days and determined the concentration of C1Q and C3 by ELISA. We observed low, but increasing levels of C1Q and C3 by day 5 (Fig. 9e), suggesting that the ovary could contribute to the local production of circulating complement proteins.

Finally, it is not surprising that the ovaries analyzed showed a pronounced population of *CD53*^high^/*CXCR4*^high^ immune cells (Fig. 2e), including separate clusters for adaptive T lymphocytes and Natural Killer (NK) cells (CL4 and CL12), B lymphocytes (CL18), and innate immune system, such as monocytes and macrophages (CL13) (Fig. 2c, d). Note that some of the innate immune cells expressed high levels of *CD68* and *IFI30*, as well as complement component C1QA (Fig. 9f, g). B lymphocytes expressed high levels of *JCHAIN* and *IGKC* (Fig. 9h) and T lymphocytes (and NK cells) expressed *IL7R*, *LTB*, *CCL5*, and *NKG7* (Fig. 9i).

## Discussion

Several studies have used single-cell technology to reveal the molecular signatures of fetal^24,25^ and adult oocytes^26–31^ during human oogenesis. Li and colleagues have investigated the molecular signature of human ovarian fetal somatic cells, including GC present in primordial follicles, providing insights in the signaling network that takes place during the formation of primordial follicles during fetal life^24^. From adult ovaries, pools of 10 GC isolated from several preantal follicles and cumulus–oocyte complex^26,30^ have been analyzed. Differences between human atretic and growing follicles (oocyte and GC) have not been reported and other somatic ovarian cells in the adult ovary have not been characterized at the transcriptional level. Our dataset provides a first step to fill the gap in knowledge regarding the characterization of the somatic cell types present in the adult ovary.

It is well accepted that the events that lead to ovulation (including remodeling of extracellular matrix, chemotaxis, microcirculatory vasomotion, formation of the oocyte–cumulus complex) are regulated by a cytokine-mediated inflammatory response orchestrated by lymphocytes, granulocytes, and macrophages^32^. Moreover, GC present in ovulatory follicles seem to have properties of innate immune cells^33^. However, the mechanisms that regulate follicular remodeling and ultimately regression are much less well understood^5,11,34,35^. Although it is feasible that the components of the complement system expressed in stroma and TC have no impact on follicular remodeling, their local production in the human ovary is intriguing. In agreement with our results, microarray analysis of TC from atretic follicles (3–5 mm diameter) also revealed a prominent up-regulation of components of the complement system (such as *C1R*, *C1S*, *C7*, *SERPING1*) when compared to healthy TC in bovine^36^. In that study, inflammatory response pathways rather than cell death characterized the atretic TC in bovine. The local activation of the complement system, contributing to an inflammatory and immune response as observed in certain organs^23^, may be also taking place in the human ovary. This may prove important for the physiological homeostasis of the ovary, perhaps potentiating follicular remodeling and its role deserves to be explored in the future.

Female infertility can be caused by immune system disorders^37,38^. Increased activation of complement system in peritoneal fluid has been associated with endometriosis-associated infertility^39^. Moreover, patients with systemic lupus erythematosus, an autoimmune disease in some cases caused by a C1Q-deficiency^40,41^, showed levels of infertility that are higher than in the normal population^42^. Polycystic ovary syndrome has been confirmed as a low-level chronic inflammation impacting on ovulation and luteinization^43,44^. Although female reproductive (dis)function has not been directly linked to the complement system, our study has identified the complement system as possible mechanism to regulate homeostasis and tissue remodeling in the adult ovary.

## Methods

### Ethical permission and collection of human material

Ovaries from cancer patients undergoing elective ovariectomy, prior to cancer treatment, were removed for the purpose of fertility preservation (cryopreservation). The phase of menstrual cycle was not determined prior to surgery. Signed informed consent was obtained form all patients to perform research on the anonymized rest material left over from the cryopreservation procedure. The research was approved by the Medical Ethical Committee of the Leiden University Medical Center (CME 05/03K/YR). After the outer layer of the ovary (1 mm thick) was collected for cryopreservation purposes and the inside of the ovary was fragmented for further analysis (single-cell RNA-sequencing or immunofluorescence).

### Single-cell dissociation of ovarian tissue

A total of 31 adult ovarian tissue samples (2–8 mm diameter) containing (whole or parts of) a single visible follicle (1–2 mm or 2–5 mm diameter) or without visible follicles were dissociated for single cell transcriptomics as previously described^45^. Briefly, individual tissue samples from adult ovary were incubated overnight on ice with 1 mg/ml Collagenase Type II (Life Technologies) in 0.25% Trypsin-EDTA (Life Technologies). Next, the samples were centrifuged at 160 × *g* for 3 min and incubated with Advanced DMEM/F12+ Glutamax (Life Technologies), 1x Insulin-Transferin-Selenium (Life Technologies), 1x Penicillin/Streptomycin (Life Technologies) and 27 IU/ml RNase-free DNase I (Qiagen) at 37 °C for 30 min to 2 h. The digestion was stopped by adding 10% of fetal calf serum (Gibco), followed by a filtration step through a 100 µm strainer (Corning). Samples were centrifuged at 160 × *g* for 5 min and stored in liquid nitrogen in Bambanker (Nippon Genetics).

### Fluorescence-activated cell sorting (FACS)

Single cells were resuspended in FACS buffer composed of 1% bovine serum albumin (BSA, Life Technologies), 2 mM EDTA (Life Technologies) in DPBS without calcium and magnesium (Life Technologies) and passed through the (pre-wet) strainer cap of FACS tubes (Corning). Cells were stained with 7-AAD (1:100, BioLegend) 3 min on ice. Live cells were sorted on a BD FACSAria I (BD Biosciences) equipped with blue laser and 695/40A long pass filter and BD FACSDiva 8.0.1 software and collected in 1% BSA in DMEM/F12 (Life Technologies) with 1x Penicillin/Streptomycin.

### RNA-sequencing and primary sequencing analysis

The library preparation was performed using the Chromium Single Cell 3′ Reagent Kit, version 2 (10X Genomics) and sequenced on a HiSeq4000 using a 300 cycles kit (Illumina). Raw sequencing data was processed using Cell Ranger analysis pipeline v2.1.1. Reads were aligned to human genome version GRCh38. For downstream analysis Cell Ranger output “filtered gene-barcode” count matrix, containing the expression profile of cells with a correctly detected cellular barcode, was used.

### Secondary sequencing analysis

For further analysis, we adapted a workflow that makes use of R package Seurat, v2.2.0^14^. The following parameters were used to filter good quality cells (and exclude cells with extreme values indicating low complexity, duplets or apoptotic cells): the total number of expressed genes/cell was 200 < nGenes < 2500; the total number of UMIs/cell was 300 < nUMIs < 15000; and the percentage of UMIs mapping to mitochondrial genes to total genes was percent.mito < 0.1. In addition, cells with more than 6% of UMIs mapping to dissociation-induced genes, as based on literature^15^, were not further analyzed and cell-cycle effects were regressed out^46^. Counts were normalized using the default normalization approach of Seurat (Function NormalizeData). Briefly, for each cell, the UMI counts for each gene were divided by the sum of UMI counts for all genes for that cell. The result was multiplied by a fixed factor (10,000) and log_e_ transformed.

To correct for patient-effects (*N* = 5) the mutual nearest neighbor (MNN) method^47^ from R package scran (v1.10), function fastMNN was used. Input for fastMNN were the 20 principal components calculated in a previous step in the workflow. The output from fastMNN, corrected for patient-effects, was used further downstream for the calculation of cell clustering and the tSNE plot. fastMNN also calculated the percentage of variance lost from each patient during orthogonalization at each merge step. The proportion of variance lost for each patient was reasonably low (P0 = 0.16, P2 = 0.04, P3 = 0.03, P7 = 0.08, P9 = 0.07).

Function FindAllMarkers from R package Seurat performed differential expression analysis (paired-wise) between the cells of a cluster and the rest of the cells in the dataset (Supplementary Data 2, 4, 5 for top 30 genes). The list of DEGs per cluster with adjusted *p*-value < 0.01 (Wilcoxon rank sum test) was used for a GO terms enrichment analysis using R packages topGO v2.30.0^48^ and org.Hs.eg.db v3.5.0^49^. The significance (*p*-value) of each GO term was estimated using the Kolmogorov–Smirnov test (Supplementary Data 3 for top 20 GO terms). Function SplitDotPlotGG from R package Seurat have been used to generate the dot plot.

For each cluster, the mean expression of all genes was calculated and the 50 most variable gene means were selected using R function rowVars, from package genefilter 1.60.0^50^. Those were used to generate a heatmap using function heatmap.2 from R package gplots v3.0.1^51^ and R function hclust was used with distance metric set to ‘manhattan’ and hierarchical clustering the agglomeration method set to ‘complete’.

To infer cell trajectories, we used two methods: one implemented in R package monocle^52^ (http://cole-trapnell-lab.github.io/monocle-release/monocle3/) and diffusion maps^53^ implemented in R package destiny. Monocle 3 alpha (v2.99.3) was used to order the cells and infer their trajectory. In this workflow, UMAP, a non-linear dimensionality reduction method, is used. UMAP parameters (n_neighbors and min_dist) values were selected to optimize the representation of cells in the two-dimensional UMAP plot. Last, the beginning of pseudotime was selected on the UMAP plot based on expression of selected markers. Diffusion map plots were calculated by running the RunDiffusion function of Seurat with default settings. RunDiffusion calls function DiffusionMap from package destiny v2.12.0.

Network analysis was generated by using GeneMANIA (http://genemania.org/) on the top 30 DEGs from the clusters of interest. Venn diagrams were generated with webtool http://bioinformatics.psb.ugent.be/webtools/Venn/ using the top200 DEGs from each cluster and gene enrichment analysis was done with Metascape (http://metascape.org/).

### Immunofluorescence

Adult ovarian tissue samples (2–8 mm diameter) containing one or several visible follicles (1–2 mm and 2–5 mm diameter) were fixed overnight in 4% paraformaldehyde at 4 °C, transferred to 70% ethanol and embedded in paraffin using a using a Shandon Excelsior tissue processor (Thermo Scientific, Altrincham, UK). Paraffin blocks were sectioned (5 μm thickness) using a RM2065 microtome (Leica Instruments GmbH, Wetzlar, Germany) onto StarFrost slides (Waldemar Knittel). For immunostaining, paraffin sections were deparafinized in xylene (2 × 10 min) followed by rehydration though a series of ethanol (100%, 100%, 90%, 80%, 70%) and ending with distilled water at room temperature (RT). For antigen retrieval, sections were treated for 20 min at 98 °C in a microwave (TissueWave 2, Thermo Scientific) with 0.01 M sodium citrate buffer (pH 6.0), except for immunostaining with rabbit anti-C1Q or goat anti-C1S that used Tris–EDTA buffer (10 mM Tris, 1 mM EDTA solution, pH 9.0). After cooling down, the slides were rinsed three times with phosphate-buffered saline (PBS) and blocked for 1 h at RT in blocking buffer (1% BSA, 0.05% Tween-20 in PBS). Subsequently, sections were incubated at RT overnight with primary antibodies, followed by 1 h with secondary antibodies, all diluted in blocking buffer. The primary antibodies used were rabbit anti-KI67 (1:100, ab15580, Abcam), mouse anti-AMH (1:30, MCA2246T, BioRad), rabbit anti-Troponin I (H-170) (1:100, sc15368, Santa Cruz), goat anti-VASA/DDX4 (1:200, AF2030, R&D), mouse anti-Cytokeratin (1:100, M351501, DAKO), rabbit anti-Wilm’s Tumor protein (1:100, CA1026-50, Calbiochem), mouse anti-StAR (D-2) (1:100, sc166821, Santa Cruz), rabbit anti-alpha smooth muscle actin/ACTA2 (1:200; ab5694, Abcam), goat anti-ZP3 (N-20) (1:100, sc23715, Santa Cruz), mouse anti-Connexin-43/GJA1 (CX-1B1) (1:50, 13-8300, Zymed), rabbit anti-Fragilis/IFITM3 (1:200, ab15592, Abcam), rabbit anti-Collagen Type IV (1:50, AB748, Chemicon), rabbit anti-Von Willebrand factor/VWF (1:100, ab6994, Abcam), mouse anti-N-Cadherin/CDH2 (GC-4) (1:100, C3865, Sigma-Aldrich), rabbit anti-PECAM1 (M-20) (1:200, sc1506, Santa Cruz), rabbit anti-c-FOS (1:20, PC38, Calbiochem), mouse anti-8-OHdG (1:1000, sc66036, Santa Cruz), goat anti-IGFBP5 (1:50, AF875, R&D), rabbit anti-C1Q (1:400, A0136, DAKO), goat anti-C1S (1:400, A302, Quidel) and mouse anti-CD68 (1:50, M087629-2, DAKO). The secondary antibodies used were Alexa Fluor 488 donkey anti-rabbit IgG (1:500, A-21206, Life Technologies), Alexa Fluor 594 donkey anti-mouse IgG (1:500, A-21203, Life Technologies), Alexa Fluor 594 donkey anti-goat IgG (1:500, A11058, Life Technologies) and Alexa Fluor 647 donkey anti-goat IgG (1:500, A-21447, Life Technologies). Cell death (TUNEL-assay) was detected by In Situ Cell Death Detection Kit (FITC) (11684817910, Sigma-Aldrich) according to the manufacturer’s instructions. Nuclei were stained with 4′,6-diamidino-2-phenyl-indole (DAPI, Life Technologies) and sections mounted using ProLong Gold (Life Technologies).

### Imaging

Immunostained slides were scanned with Pannoramic 250 Flash III digital scanner (3DHISTECH Ltd., Budapest, Hungary) and representative areas were selected for imaging using ‘Pannoramic Viewer’ (3D HISTECH, Budapest, Hungary) software.

Confocal fluorescence images were obtained on a Leica TC SP8 inverted confocal microscope (Leica) equipped with white light laser and LAS X software (Leica) or an Inverted Leica TC SP5 confocal microscope (Leica) with the LAS AF software (Leica) using a ×40 oil immersion objective (HC PL APO ×40/1.30 Oil CS2). Color adjustments was done using Fiji^54^ and single channel images shown were converted to an intensity map using Fire lookup table. Figures were assembled using Adobe Illustrator software (Adobe).

### C1Q and C3 ELISA on human ovarian tissue

Small pieces (2 × 2 × 2 mm) of ovarian stroma (*N* = 3) and atretic follicle walls (*N* = 6) from one human adult ovary were cultured individually in 96-wells plates (655180, Cellstar) with 120 µl McCoy’s 5A (Modified) Medium (22330-021, Life Technologies) supplemented with 5% fetal bovine serum (FBS, FB1001, Biosera), l-Glutamine (2 mM, 25030081, Thermo Scientific) and Penicillin–Streptomycin (50 U/ml, 15070063, Thermo Scientific). After 1 and 5 days of culture, medium (30 µl) was collected and stored at −20 °C.

The concentration of C1Q and C3 in the culture medium was determined by an in house-made ELISA^55^. Briefly, Nunc Maxisorp plates (430341, Thermo Scientific) were either coated with a solution (1 µg/ml) of mouse anti-human C1Q^56^ or rabbit anti-human anti-C3c (A0062, DAKO) diluted in coating buffer (0.1 M Na_2_CO_3_, 0.1 M NaHCO_3_) overnight at RT. After three times washing with 0.05% Tween-20 in PBS and blocking with 1% BSA in PBS for 1 h at 37 °C, the culture media diluted in PBT buffer (1% BSA, 0.05% Tween-20 in PBS) was added (1:1 dilution for C1Q and 1:12 dilution for C3) to the plates for 1 h at 37 °C. After three times washing with 0.05% Tween-20 in PBS, the plates were incubated with either rabbit anti-human C1q (1:1000, A0136, DAKO) or goat anti-human C3 (1:5000, A213, Complement technology), followed by goat anti-rabbit Ig-HRP (1:5000, P0448, DAKO), or rabbit anti-goat Ig-HRP (1:5000, P0449, DAKO), respectively, all diluted in PBT buffer. The enzymatic activity of HRP was measured after incubation with ABTS (A1888-5G, Merck) and H_2_O_2_ (1072090250, Merck) at absorbance 415 nm on a microplate reader (BioRad). The concentration of C1Q and C3 was determined by comparison to a dilution standard of normal human serum.

### Reporting summary

Further information on research design is available in the Nature Research Reporting Summary linked to this article.

## Supplementary information

Supplementary Information

Description of Additional Supplementary Files

Supplementary Data 1

Supplementary Data 2

Supplementary Data 3

Supplementary Data 4

Supplementary Data 5

Reporting Summary

## Data Availability

RNA-sequencing data are deposited in Gene Expression Omnibus (GEO) with accession number GSE118127 (https://www.ncbi.nlm.nih.gov/geo/query/acc.cgi?acc=GSE118127).

## Code availability

To ensure the reproducibility of the analysis, Conda package (version 4.6.14) management and environment management system was used (https://conda.io/docs). The workflow used for this analysis is written in R and the source code is available at github, together with the Conda environment profile (https://github.com/johnmous/singleCell).
