# Supplementary material for: Single-cell reconstruction of follicular remodeling in the human adult ovary
Source: Nat Commun. 2019 Jul 18;10:3164. doi: 10.1038/s41467-019-11036-9 (PMC6639403; doi:10.1038/s41467-019-11036-9)
Supplement: Supplementary file 1 — Supplementary Information [file 41467_2019_11036_MOESM1_ESM.pdf]

Single-cell reconstruction of follicular remodeling in the human adult ovary

FAN et al.

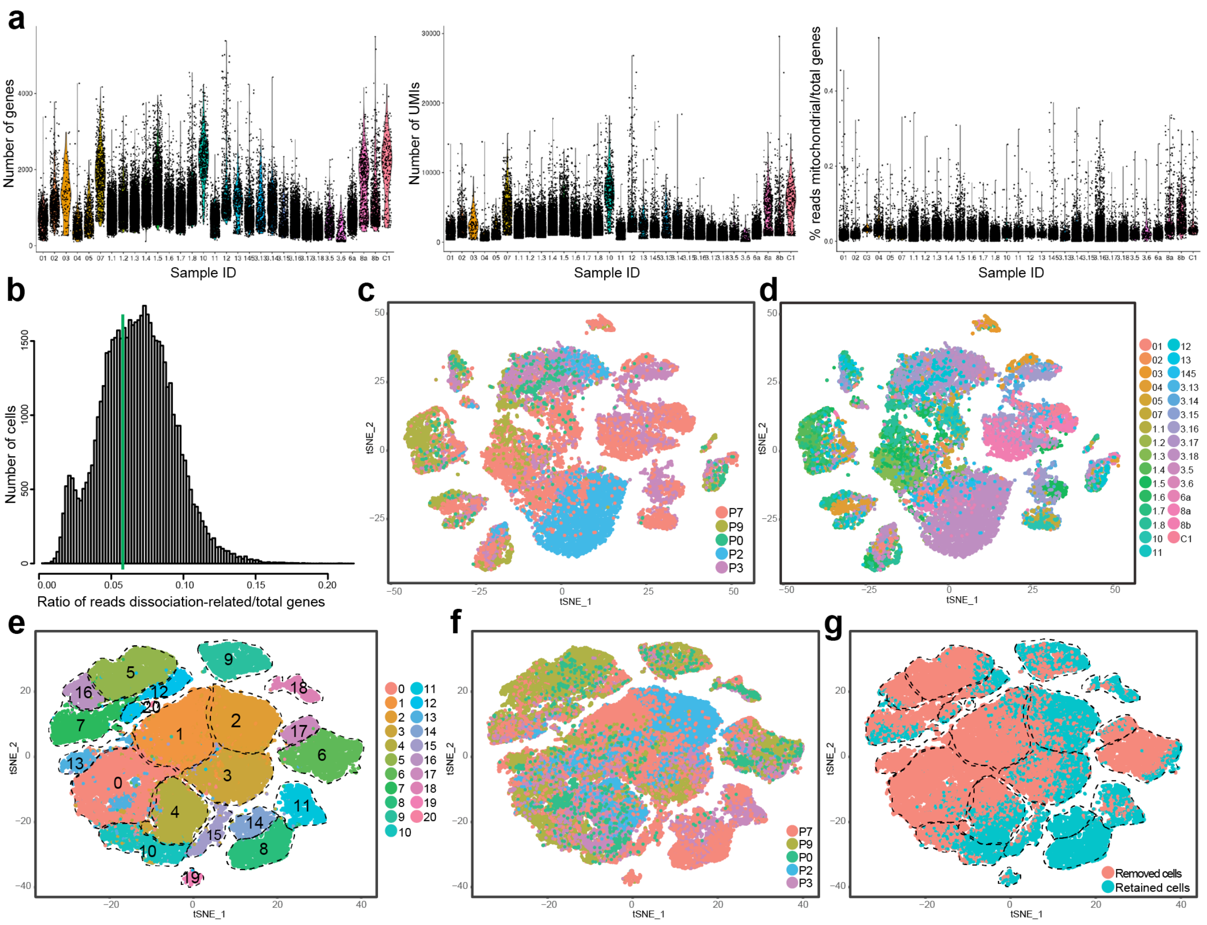

Supplementary Figure 1. Characteristics and quality control of the material used.

a, Violin plots showing the total number of genes per cell (black dots) in each sample, the total number of unique molecular identifiers (UMIs) per cell and the % reads of mitochondrial genes versus total number of genes in each sample.

b, The cut-off used (6%) regarding ratio of reads of dissociation-related genes per total reads for all genes.

c, tSNE cluster map showing the distribution of cells from each patient (P).

d, tSNE cluster map showing the distribution of cells from each sample.

e, tSNE cluster map showing the distribution of all sequenced cells. Black dashed line gives the boundaries of the clusters.

f, tSNE cluster map showing the distribution of sequenced cells colored by patient (P).

g, tSNE cluster map showing the distribution of all sequenced cells with in blue the cells that passed the quality control (retained cells) and in red the cells that were excluded from further analysis (removed cells).

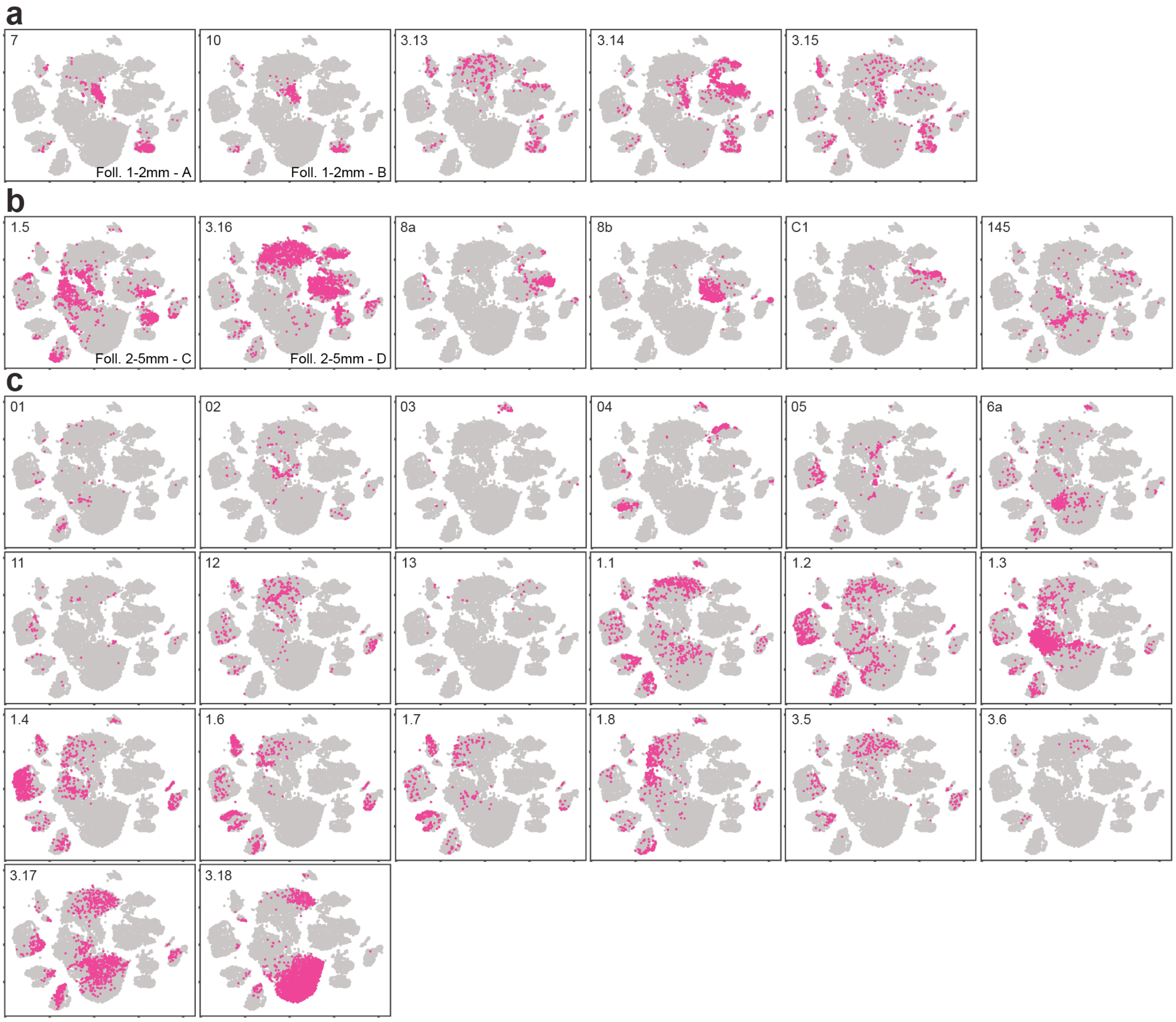

Supplementary Figure 2. Single cell distribution of each sample on the tSNE plot.

a-c, Distribution of single cells from individual samples containing (whole or parts of) a single visible follicle of 1-2mm (**a**), follicles of 2-5mm (**b**) and stroma without visible follicles (**c**) on the general tSNE plot. Follicle A, B, C are from patient P7 and Follicle D is from patient P3. Figure associated with **Figure 4a**.
