# Supplementary material for: Single-cell reconstruction of follicular remodeling in the human adult ovary
Source: Nat Commun. 2019 Jul 18;10:3164. doi: 10.1038/s41467-019-11036-9 (PMC6639403; doi:10.1038/s41467-019-11036-9)
Supplement: Supplementary file 2 — Description of Additional Supplementary Files [file 41467_2019_11036_MOESM2_ESM.pdf]

Description of Additional Supplementary Files

Supplementary Data 1: Sample characteristics and filtering steps

Supplementary Data 2: Top 30 differentially expressed genes per cluster of retained cells

Supplementary Data 3: GO analysis per cluster of retained cells

Supplementary Data 4: Top 30 differentially expressed genes per cluster of all cells (retained and discarded)

Supplementary Data 5: Top 30 differentially expressed genes per sub-cluster of theca cells (CL5)
