# Supplementary material for: Single-cell reconstruction of follicular remodeling in the human adult ovary
Source: Nat Commun. 2019 Jul 18;10:3164. doi: 10.1038/s41467-019-11036-9 (PMC6639403; doi:10.1038/s41467-019-11036-9)
Supplement: Supplementary file 8 — Reporting Summary [file 41467_2019_11036_MOESM8_ESM.pdf]

natureresearch

Corresponding author(s): S.M. Chuva de Sousa Lopes
lopes@lumc.nl

Reporting Summary

Nature Research wishes to improve the reproducibility of the work that we publish. This form provides structure for consistency and transparency in reporting. For further information on Nature Research policies, see Authors & Referees and the Editorial Policy Checklist.

Statistical parameters

When statistical analyses are reported, confirm that the following items are present in the relevant location (e.g. figure legend, table legend, main text, or Methods section).

n/a	Confirmed	
☐	☒	The exact sample size (*n*) for each experimental group/condition, given as a discrete number and unit of measurement
☐	☒	An indication of whether measurements were taken from distinct samples or whether the same sample was measured repeatedly
☐	☒	The statistical test(s) used AND whether they are one- or two-sided<br>*Only common tests should be described solely by name; describe more complex techniques in the Methods section.*
☒	☐	A description of all covariates tested
☐	☒	A description of any assumptions or corrections, such as tests of normality and adjustment for multiple comparisons
☐	☒	A full description of the statistics including central tendency (e.g. means) or other basic estimates (e.g. regression coefficient) AND variation (e.g. standard deviation) or associated estimates of uncertainty (e.g. confidence intervals)
☐	☒	For null hypothesis testing, the test statistic (e.g. *F*, *t*, *r*) with confidence intervals, effect sizes, degrees of freedom and *P* value noted<br>*Give P values as exact values whenever suitable.*
☒	☐	For Bayesian analysis, information on the choice of priors and Markov chain Monte Carlo settings
☐	☒	For hierarchical and complex designs, identification of the appropriate level for tests and full reporting of outcomes
☒	☐	Estimates of effect sizes (e.g. Cohen's *d*, Pearson's *r*), indicating how they were calculated
☒	☐	Clearly defined error bars<br>*State explicitly what error bars represent (e.g. SD, SE, CI)*

Our web collection on statistics for biologists may be useful.

Software and code

Policy information about availability of computer code

Data collection	FACS software: BD FACS Diva 8.0.1, Image acquisition software: Leica LAS and Leica LAX
Data analysis	Image J 2.0.0-rc-43/1.5p bundled with Java 8_117 for image processing; Adobe Illustrator CS6 for figure assembly; The Cell Ranger Single Cell Software Suite version 2.1.1 for sample demultiplexing, barcode processing, read aligning and single cell 3' gene counting using the Illumina BCL files as input. scRNA-Seq data analysis was performed using scripts in R (available on request).

For manuscripts utilizing custom algorithms or software that are central to the research but not yet described in published literature, software must be made available to editors/reviewers upon request. We strongly encourage code deposition in a community repository (e.g. GitHub). See the Nature Research guidelines for submitting code & software for further information.

Data

Policy information about availability of data

All manuscripts must include a data availability statement. This statement should provide the following information, where applicable:

- Accession codes, unique identifiers, or web links for publicly available datasets
- A list of figures that have associated raw data
- A description of any restrictions on data availability

All sequencing data are available in the Gene Ontology Omnibus repository under the accession number GSE118127

Field-specific reporting

Please select the best fit for your research. If you are not sure, read the appropriate sections before making your selection.

☒ Life sciences ☐ Behavioural & social sciences ☐ Ecological, evolutionary & environmental sciences

For a reference copy of the document with all sections, see nature.com/authors/policies/ReportingSummary-flat.pdf

Life sciences study design

All studies must disclose on these points even when the disclosure is negative.

Sample size	No statistical method was used to predetermine the sample size. Sample size used for scRNA-Seq was N=5 for the number of human individuals. Total samples from different ovarian regions submitted for scRNA-Seq was N=31. Data validation by immunostainig was performed on the same individuals (N=5).
Data exclusions	As a part of the analysis cells with high count of mitochondial genes were excluded from further analysis as well as cells with few UMI counts and genes (see methods and extended data 1). Moreover, cells with more than 6% of of the total UMI counts from dissociation-genes were discarded from further analysis (see methods and extended data 1).
Replication	scRNA-Seq was done in two independent sequencing runs and yielded similar results, immunostainings were performed in technical triplicates.
Randomization	Human samples were used without randomization from patients undergoing ovariectomy as a part of the fertility preservation treatment.
Blinding	All samples were processed with identical computational pipelines, therefore blinding was not used.

Reporting for specific materials, systems and methods

Materials & experimental systems

n/a	Involved in the study
☐	☒ Unique biological materials
☐	☒ Antibodies
☒	☐ Eukaryotic cell lines
☒	☐ Palaeontology
☒	☐ Animals and other organisms
☒	☐ Human research participants

Methods

n/a	Involved in the study
☒	☐ ChIP-seq
☐	☒ Flow cytometry
☒	☐ MRI-based neuroimaging

Unique biological materials

Policy information about availability of materials

Obtaining unique materials	Human ovarian tissue samples were obtained from patients undergoing ovariectomy for fertility preservation, previous to cancer treatment. The material used in the study is considered rest material and is further not used for fertility preservation. Signed informed consent was obtained from the patients according to EU and national regulations.

Antibodies

Antibodies used

Flow cytometry: 7AAD (420403) BioLegend.
TUNEL: Cell death (TUNEL-assay) was detected by In Situ Cell Death Detection Kit (FITC) (11684817910, Sigma-Aldrich) according to the manufacturer's instructions.
ELISA: rabbit anti-human anti-C3c (A0062, DAKO), mouse anti-human C1Q, rabbit anti-human C1q (A0136, DAKO), goat anti-human C3 (A213, Complement technology); goat anti-rabbit Ig-HRP (P0448, DAKO) or rabbit anti-goat Ig-HRP (P0449, DAKO)
Immunofluorescence:
nuclear counterstain DAPI (D9542) Sigma-Aldrich;
secondary antibodies: Alexa Fluor 488 donkey anti rabbit IgG (A21206), Alexa Fluor 594 donkey anti mouse IgG (A21203), Alexa Fluor 594 donkey anti goat IgG (A11058), Alexa Fluor 647 donkey anti goat IgG (A21447) purchased from Life Technologies.
primary antibodies:
mouse anti-CD68 (1:50, M087629-2, DAKO),rabbit anti-c-FOS (1:20, PC38, Calbiochem), mouse anti-8-OHdG (1:1000, sc66036, Santa Cruz), goat anti-IGFBP5 (1:50, AF875, R&D),rabbit anti-ki67 (1:100, ab15580, Abcam),Mouse anti-AMH (MCA2246T) Bio-

Rad, Rabbit anti-Troponin I (sc-15368) Santa Cruz, Goat anti-DDX4 (AF2030) R&D, Mouse anti-Cytokeratin (M351501) DAKO, Rabbit anti-Wilms Tumor 1 (CA1026-50) Calbiochem, Mouse anti-StAR (sc-166821) Santa Cruz, Rabbit anti-Alpha smooth muscle Actin (ab5694) Abcam, Goat anti-ZP3 (sc-23715) Santa Cruz, Mouse anti-Connexin 43 (13-8300) Zymed, Rabbit anti-Fragilis (ab15592) Abcam, Rabbit anti-Collagen type IV (AB748) Chemicon, Rabbit anti-Von Willebrand Factor (vWF) LifeSpan (ab6994) Biosciences, C1q Rabbit (A0136) DAKO, C1s Goat (A302) Quidel

Validation: All antibodies used are commercially available and have been validated by the manufacturer, except for mouse anti-human C1Q

Flow Cytometry

Plots

Confirm that:

☒ The axis labels state the marker and fluorochrome used (e.g. CD4-FITC).

☐ The axis scales are clearly visible. Include numbers along axes only for bottom left plot of group (a 'group' is an analysis of identical markers).

☐ All plots are contour plots with outliers or pseudocolor plots.

☐ A numerical value for number of cells or percentage (with statistics) is provided.

Methodology

Sample preparation	Tissue was cut in small pieces and treated overnight in 1mg/ml colII in 0.25% Trypsin/EDTA at 4C. Next day, it was incubated 1h at 37C in DMEM/F12 with DNAseI (27 IU/ml), filtered through a 100um strainer and single cells were cryopreserved. Before library preparation, cells were thawed, ressuspended in 1% BSA/DPBS and FACS-sorted for live cells (7AAD-negative cells).
Instrument	BD FACSAria1
Software	BD FACS Diva 8.0.1
Cell population abundance	Cells were sorted based on 7AAD vital stain. Cell that did not incorporate the dye (live cells) were sorted out and used for scRNA-Seq. Negative populations differed between samples and ranged from 3-73.4% of total cells.
Gating strategy	Cells were gated based on FSC-A/SSC-A pattern for P1, then FSC-W/FSC-H (P2) and SSC-W/SSC-H (P3) for singlets. 7AAD negative population was sorted with long pass filter 695/40-A. Gating strategy in Figure 2A.

☒ Tick this box to confirm that a figure exemplifying the gating strategy is provided in the Supplementary Information.
